# Hemagglutinin and Neuraminidase Antibodies Are Induced in an Age- and Subtype-Dependent Manner after Influenza Virus Infection

**DOI:** 10.1128/JVI.01385-19

**Published:** 2020-03-17

**Authors:** Sook-San Wong, Ben Waite, Jacqui Ralston, Tim Wood, G. Edwin Reynolds, Ruth Seeds, E. Claire Newbern, Mark G. Thompson, Q. Sue Huang, Richard J. Webby

**Affiliations:** a State Key Laboratory of Respiratory Disease, Guangzhou Medical University, Guangzhou, People’s Republic of China; b School of Public Health, Li Ka Shing Faculty of Medicine, The University of Hong Kong, Hong Kong, Hong Kong SAR, People’s Republic of China; c Department of Infectious Diseases, St. Jude Children’s Research Hospital, Memphis, Tennessee, USA; d Institute of Environmental Science and Research, Ltd., NCBID–Wallaceville, Wallaceville, New Zealand; e Immunisation Advisory Centre (IMAC), University Services, University of Auckland, Auckland, New Zealand; f Influenza Division, Centers for Disease Control and Prevention, Atlanta, Georgia, USA; Icahn School of Medicine at Mount Sinai

**Keywords:** influenza, antibody, hemagglutination inhibition, neuraminidase inhibition, serology

## Abstract

Despite evidence that antibodies targeting the influenza virus neuraminidase (NA) protein can be protective and are broadly cross-reactive, the immune response to NA during infection is poorly understood compared to the response to hemagglutinin (HA) protein. As such, we compared the antibody profile to HA and NA in two naturally infected human cohorts in Auckland, New Zealand: (i) a serosurvey cohort, consisting of pre- and post-influenza season sera from PCR-confirmed influenza cases (*n* = 50), and (ii) an immunology cohort, consisting of paired sera collected after PCR-confirmation of infection (*n* = 94). The induction of both HA and NA antibodies in these cohorts was influenced by age and subtype. Seroconversion to HA was more frequent in those <20 years old (yo) for influenza A (serosurvey, *P* = 0.01; immunology, *P* = 0.02) but not influenza B virus infection. Seroconversion to NA was not influenced by age or virus type. Adults ≥20 yo infected with influenza A viruses were more likely to show NA-only seroconversion compared to children (56% versus 14% [5 to 19 yo] and 0% [0 to 4 yo], respectively). Conversely, children infected with influenza B viruses were more likely than adults to show NA-only seroconversion (88% [0 to 4 yo] and 75% [5 to 19 yo] versus 40% [≥20 yo]). These data indicate a potential role for immunological memory in the dynamics of HA and NA antibody responses. A better mechanistic understanding of this phenomenon will be critical for any future vaccines aimed at eliciting NA immunity.

**IMPORTANCE** Data on the immunologic responses to neuraminidase (NA) is lacking compared to what is available on hemagglutinin (HA) responses, despite growing evidence that NA immunity can be protective and broadly cross-reactive. Understanding these NA responses during natural infection is key to exploiting these properties for improving influenza vaccines. Using two community-acquired influenza cohorts, we showed that the induction of both HA and NA antibodies after infection is influenced by age and subtypes. Such response dynamics suggest the influence of immunological memory, and understanding how this process is regulated will be critical to any vaccine effort targeting NA immunity.

## Abstract (precis)

Data on the immunologic responses to neuraminidase (NA) is lacking compared to what is available on hemagglutinin (HA) responses, despite growing evidence that NA immunity can be protective and broadly cross-reactive. Understanding these NA responses during natural infection is key to exploiting these properties for improving influenza vaccines. Using two community-acquired influenza cohorts, we showed that the induction of both HA and NA antibodies after infection is influenced by age and subtypes. Such response dynamics suggest the influence of immunological memory, and understanding how this process is regulated will be critical to any vaccine effort targeting NA immunity.

## INTRODUCTION

Neuraminidase (NA) is the second most abundant glycoprotein on the surface of the influenza virus. It functions as a sialidase to facilitate virus trafficking through host mucosal barriers, as well as egress from infected cells. Inhibition of its activity has been the cornerstone of currently recommended influenza antivirals (https://www.cdc.gov/mmwr/pdf/rr/rr6001.pdf). Although immunity to NA limits disease severity (1–4), relatively little is known about population immunity to the protein or the robustness of the antibody response against it following infection. These facets of NA immunity have been identified as crucial knowledge gaps in the campaign to develop better seasonal influenza vaccines (5–7).

The paucity of data on NA immunity has historically been impeded by the lack of an appropriate and scalable assay. The traditional NA inhibition (NAI) assay that was based on the enzymatic cleavage of sialic acids on the substrate fetuin was laborious and required the use of hazardous chemicals such as arsenite and thiobarbituric acid (8). However, the development and validation of an enzyme-linked lectin assay (ELLA) has enabled rapid and high-throughput testing of NAI antibodies (9, 10), leading to renewed interest in evaluating the role of NA antibodies during influenza virus infection and vaccination (1, 2, 4, 11, 12). The ELLA has shown high subtype specificity and reproducibility when tested with antigen-specific ferret antisera (9) and higher sensitivity compared to the traditional NAI assay (13).

**FIG 1:**
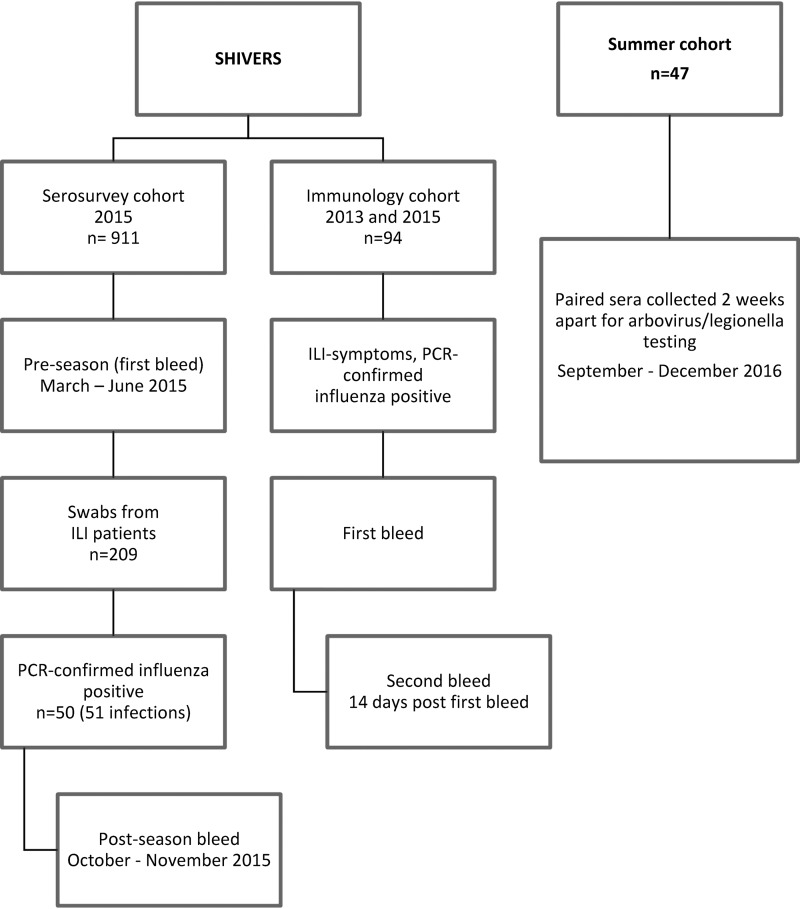
Sampling and recruitment of individuals in each study cohort.

In this study, we explored the antibody response to HA and NA following community-acquired influenza virus infection in two cohorts enrolled through the Southern Hemisphere Influenza Vaccine Effectiveness Research Surveillance (SHIVERS) study based in Auckland, New Zealand (14) (Fig. 1). The cohorts utilized distinct sampling protocols that were representative of epidemiological and clinical studies. We examined the seroconversion frequencies to HA using the standard hemagglutination inhibition (HAI) assay and NA using ELLA in individuals with PCR-confirmed influenza virus infections. Since recent studies suggest that the HAI assay may have limited sensitivity in detecting recent influenza virus infections (15–17), we further examined the added benefit of using NAI antibody responses as an alternative serologic marker of infection. Data from this study suggest that the dynamics of antibody responses to both HA and NA after infection are influenced by age and subtype and that the use of the ELLA to detect NAI antibodies after infection can circumvent some of the current limitations associated with HAI assays.

## RESULTS

### Cohort characteristics.

Table 1 presents the characteristics of the study participants and sampling details in the two cohorts of this study. The 50 PCR-confirmed influenza positive serosurvey cohort participants (one participant was infected with both IAV and IBV) were relatively equally distributed across the three age groups (< 5 years old [yo], 5 to 19 yo, and ≥20 yo). Twenty-one (42%) participants identified as Maori (*n* = 7), Pacific (*n* = 5), or of Asian (*n* = 9) descent, and the remaining participants were of European descent (classified as “others”) (Table 1). There were equal proportions of IAV (25/51, 49%) and IBV (26/51, 51%), and both B lineages were equally represented (B/Vic, 15/51, 29%; B/Yam, 11/51, 22%). The median time from preseason serum collection to symptom onset was 134 days (approximately 4 months; range, 22 to 193 days). The median interval between the preseason and postseason serum collections was approximately 7 months (218 days; range, 132 to 274 days).

**TABLE 1:** Characteristics of participants, influenza infections, and timing of serum collections in the serosurvey, immunology, and summer cohorts^*a*^

Patient characteristics or time parameters	Serosurvey cohort (*n* = 50)		Immunology cohort (*n* = 94)		Summer cohort (*n* = 47)	
	*n*	%	*n*	%	*n*	%
Age (yr)						
0–4	16	32	7	7	0	0
5–19	15	30	5	5	1	2
20+	19	38	82	87	46	98
Sex						
Male	16	32	44	47		
Female	34	68	50	53		
Ethnicity						
Maori	7	14	15	16		
Pacific	5	10	23	25		
Asian	9	18	8	9		
European descent/other	29	58	47	51		
Infection subtype						
A (not subtyped)	10	20	15	16		
A (H3)	15	29	53	56		
A (H1)	0	0	1	1		
B (not subtyped)	0	0	2	2		
B (Victoria)	15	29	4	4		
B (Yamagata)	11	22	19	20		
						
Median days (range)						
From first serum collection to symptom onset	134 (22–193), *n* = 39				
From symptom onset to first serum collection			12 (1–28), *n* = 58			
From first serum collection to swab specimen	141 (28–202)					
From swab specimen to first sera collection			6 (0–23), *n* = 93			
Between serum collections	218 (132–274)		16 (5–27), *n* = 93			

a *n*, Number of PCR-confirmed influenza cases. For the serosurvey cohort (*n* = 50 individuals), one subject was positive for influenza A and B. For the immunology cohort, ethnicity data were only available for 93 participants.

In the immunology cohort, 82 of 94 (87%) paired sera were collected from adults (≥20 yo). Forty-six (49%) participants identified as Pacific (*n* = 23), Maori (*n* = 15), or of Asian (*n* = 8) descent, and the remaining 47 were of European descent. Most infections were due to influenza A viruses (IAVs; 74%) or influenza B viruses (IBVs) of Yamagata lineage (20%). Because PCR testing and serum collection was conducted only following presentation with symptoms, the median time from symptom onset to first serum collection was 12 days (range, 1 to 28).

### HAI and NAI-antibody responses in the serosurvey cohort.

**FIG 2:**
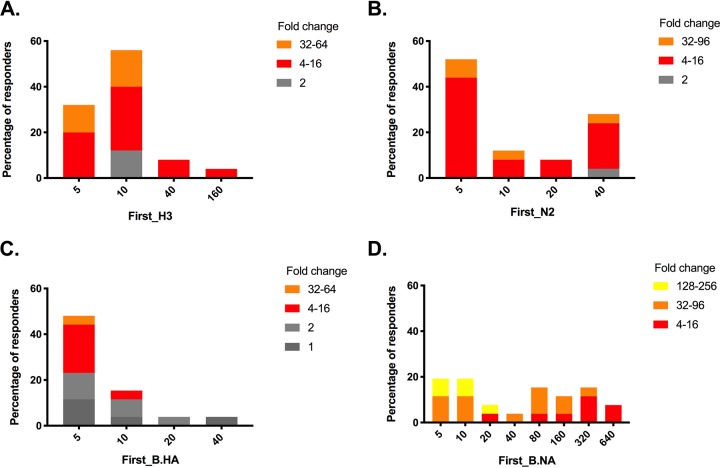
Relationship between preseason hemagglutination inhibition (HAI) (A and C) and neuraminidase-inhibition (NAI) (B and D) antibody titers with titer fold increase in postseason sera in the serosurvey cohort. (A and B) A/H3N2 cases; (C and D) influenza B cases. The *x* axes indicate the baseline HA or NAI titers, and the *y* axes indicate the percentages of responders showing the antibody fold increase according to the range indicated in the legend. The range is indicated as the fold change in titers: gray indicates titer changes of ≤2-fold, while red, orange, and yellow indicate titer changes of ≥4-fold.

The serosurvey cohort allowed us to collect true baseline samples prior to substantial community influenza activity. HAI and NAI antibody titers were determined on pre- and postseason serum samples from individuals with laboratory-confirmed influenza during the season. For participants with IAV infections (Table 2), the baseline HAI geometric mean titers (GMTs) were low in this cohort across all age groups, whereas there were relatively small increases in baseline NAI GMTs with increasing age (the GMTs and 95% confidence intervals [95% CI] for the 0 to 4, 5 to 19, and ≥20 yo groups were 5 [5 to 5], 24 [15 to 40], and 13 [7 to 24], respectively; *P* = 0.0005). The geometric mean fold change (GMFC) between the pre- and postseason sera decreased with increasing age for HAI (GMFC [95% CI] for the 0 to 4, 5 to 19, and ≥20 yo groups were 22 [14 to 35], 10 [8 to 12], and 4 [3 to 6], respectively; *P* = 0.0005) but not for NAI. There were no age-dependent differences on the NAI GMFC responses. Baseline titers did not appear to influence the magnitude of fold change for the HAI or NAI antibody titers (Fig. 2A and B).

**TABLE 2:** HAI and NAI antibody responses to influenza A and B in subjects with PCR-confirmed influenza in the serosurvey cohort

Age (yr)	Antibody titer^*a*^	Influenza A								Influenza B							
		*n*	HAI	95% CI	*P* for age	NAI	95% CI	*P* for age	By HAI or NAI	*n*	HAI	95% CI	*P* for age	NAI	95% CI	*P* for age	By HAI or NAI
0–4	GMT 1	9	7	5–9		5	5			8	8	5–13		11	7–18		
	GMT 2		148	97–226		59	31–112				15	9–27		761	435–1,332		
	GMFC		22	14–35		12	10–14				2	0–4		70	68–72		
	%SC		100	100–100		67	28–105		100		13	–17–42		100	100–100		100
5–19	GMT 1	7	14	6–31	0.1882^*b*^	24	15–40	0.0005^*b*^		8	5	5	0.1874^*b*^	31	11–86	0.0007^*b*^	
	GMT 2		131	46–378	0.1064^*c*^	238	124–457	0.0282^*c*^			22	11–44	0.2988^*c*^	1522	683–3,393	0.0520^*c*^	
	GMFC		10	8–12	0.0005^*d*^	10	8–12	0.6408^*d*^			4	2–6	0.1248^*d*^	49	47–51	0.0251^*d*^	
	%SC		86	51–121	0.0133^*e*^	100	100–100	0.1898^*e*^	100		25	–14–64	0.0905^*e*^	100	100–100	1.0000^*e*^	100
20+	GMT 1	9	12	7–19		13	7–24			10	6	5–8		149	64–348		
	GMT 2		50	23–110		101	53–191				33	17–64		2744	1,383–5,445		
	GMFC		4	3–6		8	6–10				5	3–7		18	17–20		
	%SC		44	4–85		89	63–115		100		60	23–97		100	100–100		100

a GMT, geometric mean titer; GMFC, geometric mean fold change. The percent seroconversion (%SC) was determined as the percentage of individuals that seroconverted, defined by a 4-fold rise in antibody titer in the specified assay between the first and second sera.

b Age versus GMT 1 by ANOVA.

c Age versus GMT 2 by ANOVA.

d Age versus GMFC by ANOVA.

e Age versus the percent seroconversion by ANOVA.

We next looked at seroconversion events detected by each assay, as determined by a ≥4-fold increase in titer and meeting the minimum threshold titer of 40. The frequency of HAI seroconversion in IAV was lowest in adults (44% [95% CI = 4 to 85%]) compared to children (86% [95% CI = 51 to 121%]) and young children (100%) (*P* = 0.0133). In contrast, the frequencies of NAI seroconversion ranged from 67 to 100% and were not statistically different by age.

For IBV, the baseline GMT increased significantly with age for NAI (the GMTs [95% CI] for 0 to 4, 5 to 19, and ≥20 yo subjects were 11 [7 to 18], 31 [11 to 86], and 149 [64 to 348], respectively; *P* < 0.001] but not for HAI, which was relatively low in all age groups. HAI seroconversion to IBV was more common in adults than in younger participants (the percent seroconversion [95% CI] for the 0 to 4, 5 to 19, and ≥20 yo groups were 13 [–17 to 42], 25 [–14 to 64], and 60 [23 to 97], respectively), although this had weak statistical support (*P* = 0.09). NAI seroconversion was detected in 100% of the IBV-infected individuals, with children having a larger GMFC compared to adults. Although the magnitude of the response was greater in those with low baseline NAI titers (<40), individuals with high baseline titers (between 160 and 640) were still capable of mounting at least a 4-fold increase in titer (Fig. 2C and D).

**FIG 3:**
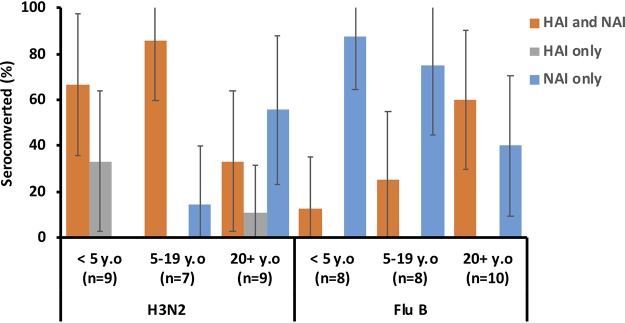
Percentages of individuals that seroconverted as determined by HAI assay only, NAI assay only, or both HAI and NAI assays in PCR-confirmed influenza cases in the serosurvey cohort. Error bars indicate 95% confidence intervals.

We next looked at seroconversion events to HA and NA at an individual level. In the serosurvey cohort, concurrent HAI and NAI seroconversions for IAV occurred in 67% (6/9), 86% (6/7), and 33% (3/9) of the 0 to 4, 5 to 19, and ≥20 yo subjects (Fig. 3), respectively. HAI seroconversions only (no NAI seroconversions) occurred in 16% (4/25) (3 in 0 to 4 yo subjects and 1 in ≥20 yo subjects) of the PCR-positive cases. NAI-only seroconversions (no HAI seroconversions) occurred in 24% (6/25) of individuals, mostly in adults (0%, 14% [1/7], and 56% [5/9] in the respective age groups). In contrast to the trend observed for IAV, the HAI and NAI seroconversions in IBV-infected individuals occurred in 12.5% (1/8), 25% (2/8), and 60% (6/10) of 0 to 4, 5 to 19, and ≥20 yo subjects, respectively. In addition, 17/26 of the IBV-infected individuals that did not show HAI seroconversion, seroconverted to NA (88% (7/8), 75% (6/8), and 40% (4/10) in the 0 to 4, 5 to 19, and ≥20 yo groups, respectively). No HAI-only seroconversions were detected.

### HAI and NAI antibody responses in the immunology cohort.

**FIG 4:**
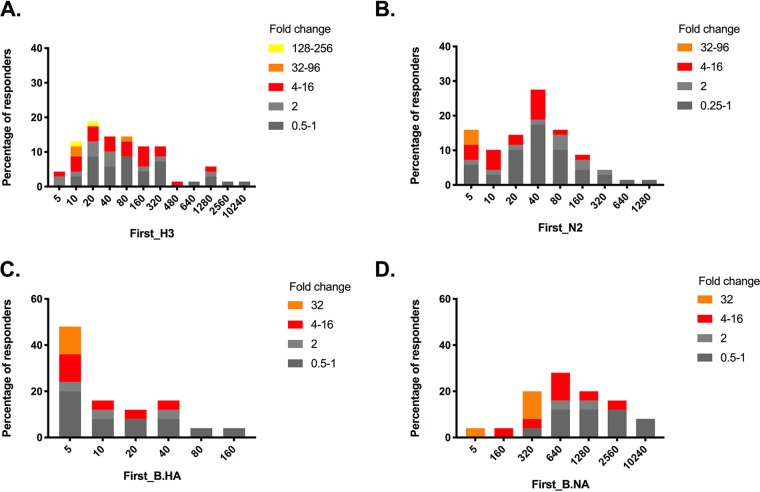
Relationship between baseline first HAI (A and C) and NAI (B and D) titers with the antibody titer fold increase in the second sera in the immunology cohort. (A and B) A/H3N2 cases; (C and D) influenza B cases. The *x* axes indicate the baseline HA or NAI titers, and the *y* axes indicate the percentages of responders showing an antibody fold increase according to the range indicated in the legend. The range is indicated as the fold change in titers: gray indicates titer changes of ≤2-fold, while red, orange, and yellow indicate titer changes of ≥4-fold.

The challenge of conducting immunologic studies in medical care facilities is that infections, as well as the corresponding immune responses, are often well under way by the time patients present. Therefore, true baseline blood draws are difficult to obtain. This was likely the case in our immunology cohort, since the baseline antibody GMTs to HA and NA were higher compared to the serosurvey cohort across all age groups (Table 3). Within this cohort there were no statistical differences in the IAV baseline GMT for HAI or NAI among the different age groups, but there were age-specific differences in the GMFC responses (HAI GMFC, *P* = 0.0014; NAI GMFC, *P* = 0.067). Subjects <5 yo showed the highest GMFC in both assays. The frequency of HAI seroconversions was highest among young children (0 to 4 yo; 83% [95% CI = 41 to 126%]) compared to adults (≥ 20 yo; 34% [95% CI = 22 to 46%]), while NAI seroconversions did not show any age-dependent trends. Unlike the serosurvey cohort, a large percentage of IAV cases across a broad range of baseline HAI and NAI titers failed to mount at least a 4-fold increase in antibody titers (Fig. 4A and B). An exception was the NAI response against IBV, where nonresponders (FC ≤ 2) had high baseline (>320) NAI titers (Fig. 4C and D).

**TABLE 3:** HAI and NAI antibody responses to influenza A and B in subjects with PCR-confirmed influenza in the immunology cohort

Age (yrs)	Antibody titer^*a*^	Influenza A								Influenza B							
		*n*	HAI	95% CI	*P* for age	NAI	95% CI	*P* for age	By HAI or NAI	*n*	HAI	95% CI	*P* for age	NAI	95% CI	*P* for age	By HAI or NAI
0–4	GMT 1	6	86	12–609		36	5–247			1	10	NA		5	NA		
	GMT 2		1220	401–3,714		160	36–709				10	NA		160	NA		
	GMFC		14	3–73		5	1–20				0	NA		32	NA		
	%SC		83	41–126		50	–8–108		83		0	NA		100	NA		100
5–19	GMT 1	4	320	184–557	0.1262^*b*^	34	24–47	0.9960^*b*^		1	20	NA	0.8833^*b*^	2560	NA	0.0002^*b*^	
	GMT 2		226	153–335	0.0185^*c*^	28	19–42	0.1067^*c*^			10	NA	0.5437^*c*^	2560	NA	0.0483^*c*^	
	GMFC		1	1	0.0014^*d*^	1	1	0.0673^*d*^			1	NA	0.4127^*d*^	1	NA	0.1885^*d*^	
	%SC		0	0–0	0.0156^*e*^	0	0–0	0.2082^*e*^	0		0	NA	0.5760^*e*^	0	NA	0.3368^*e*^	0
20+	GMT 1	59	59	40–88		34	25–46			23	11	7–18		1280	815–2,011		
	GMT 2		144	91–227		55	39–77				32	18–58		3258	2063–5145		
	GMFC		2	2–3		2	1–2				3	2–5		3	1–5		
	%SC		34	22–46		25	14–37		44		39	18–61		35	14–56		48

a GMT, geometric mean titer; GMFC, geometric mean fold change. The percent seroconversion (%SC) was determined as the percentage of individuals that seroconverted, as defined by a 4-fold rise in antibody titer by the specified assay between the first and second sera.

b Age versus GMT 1 by ANOVA.

c Age versus GMT 2 by ANOVA.

d Age versus GMFC by ANOVA.

e Age versus the percent seroconversion by ANOVA.

**FIG 5:**
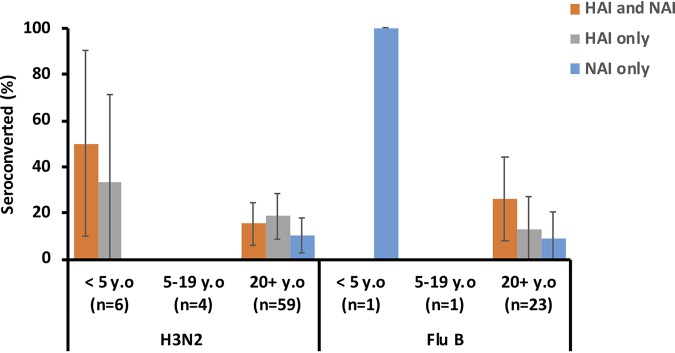
Percentages of individuals that seroconverted as determined by HAI assay, NAI assay, or both HAI and NAI assays in PCR-confirmed influenza cases in the immunology cohort. Error bars represent 95% confidence intervals.

Concurrent HAI and NAI seroconversions to IAV were marginally more frequent in subjects <5 yo (3/6, 50%) than in adults (9/59, 15%; *P* = 0.07, Fisher exact test) (Fig. 5). HAI-only seroconversion only was observed in 13/69 (19%) of individuals, whereas NAI-only seroconversions were only observed in 6/69 (9%) participants.

Age-dependent observations were limited in IBV cases due to low enrollment in the <20 yo age bracket. In subjects ≥20 yo, 39% (95% CI = 18 to 61%) and 35% (95% CI = 14 to 56%) seroconverted as determined by HAI and NAI assays, respectively. Concurrent HAI and NAI seroconversions were detected in only 6/25 (24%) individuals, while HAI- or NAI-only seroconversion occurred in 3/25 (12%) individuals, respectively.

### NAI antibodies as serologic markers of influenza virus infections.

Given the poor detection of HAI seroconversion in certain age groups, we sought to determine whether the NAI antibody responses could be used as an alternative serological marker to detect recent infections. We first determined the overall seroconversion frequencies in both cohorts by either assay or both assays, and we then evaluated the performance of these assays using a statistical metric.

In the serosurvey cohort, the overall (IAV and IBV) frequencies of HAI or NAI seroconversion events were 55% (95% CI = 41 to 69%, *n* = 28) and 92% (95% CI = 85 to 99%, *n* = 47), respectively. In the immunology cohort, the overall frequencies of HAI or NAI seroconversion events were 36% (95% CI = 26 to 46%, *n* = 34) and 28% (95% CI = 19 to 37%, *n* = 27), respectively. However, using either assay to define seroconversions captured 100 and 46% of all the infected cases in the serosurvey and immunology cohorts, respectively. Thus, including NAI seroconversions can improve the accuracy of the serologic detection of recent influenza virus infections.

### Interassay diagnostic agreement with the PCR positivity and sensitivity and the specificity of serologic assays.

We used κ statistics to evaluate the level of agreement between PCR subtype positivity and serologic seroconversions by each assay when used alone and in combination (Table 4). Higher κ scores (scaled from 0 to 1) indicate a better agreement between the two methods (18).

**TABLE 4:** Kappa (κ) agreement and test sensitivity scores for HAI and NAI assays in detecting influenza A and B infections in PCR-confirmed influenza cases^*a*^

Cohort	Assay	Influenza A (H3N2) (95% CI) score			Influenza B (95% CI) score		
		κ	Sensitivity (%)	Specificity (%)	κ	Sensitivity (%)	Specificity (%)
Serosurvey	HAI	0.48 (0.33–0.64)	0.76 (0.55–0.91)	0.87 (0.81–0.92)	0.24 (0.06–0.42)	0.35 (0.17–0.56)	0.90 (0.85–0.94)
	NAI	0.47 (0.32–0.61)	0.84 (0.64–0.95)	0.84 (0.77–0.89)	0.56 (0.43–0.69)	1.00 (0.81–1.00)	0.83 (0.77–0.89)
	HAI and NAI	0.45 (0.28–0.63)	0.60 (0.39–0.79)	0.91 (0.86–0.94)	0.28 (0.10–0.47)	0.35 (0.17–0.56)	0.92 (0.87–0.96)
	HAI or NAI	0.48 (0.36–0.62)	1.00 (0.80–1.00)	0.80 (0.73–0.85)	0.53 (0.40–0.66)	1.00 (0.81–1.00)	0.82 (0.75–0.87)
Immunology	HAI	0.27 (0.14–0.41)	0.35 (0.24–0.48)	0.92 (0.83–0.97)	0.48 (0.28–0.69)	0.35 (0.16–0.57)	0.99 (0.95–1.00)
	NAI	0.24 (0.12–0.35)	0.26 (0.17–0.39)	0.97 (0.90–1.00)	0.48 (0.28–0.68)	0.39 (0.20–0.61)	0.97 (0.93–0.99)
	HAI and NAI	0.18 (0.08–0.27)	0.18 (0.09–0.29)	1.00 (0.93–1.00)	0.34 (0.13 0.55)	0.22 (0.07–0.44)	0.99 (0.95–1.00)
	HAI or NAI	0.33 (0.19–0.47)	0.44 (0.32–0.57)	0.89 (0.79–0.95)	0.60 (0.41–0.78)	0.52 (0.31–0.73)	0.97 (0.93–0.99)

a The strength of agreement is based on the Landis and Koch scale [18]: <0.2 = poor or slight, ≥0.2 to <0.4 = fair, 0.4 to <0.6 = moderate, 0.6 to <0.8 = substantial, and 0.8 to 1 = almost perfect to perfect.

The κ scores calculated for IAV cases for PCR versus HAI alone and PCR versus NAI alone in both cohorts were comparable (serosurvey κ [95% CI] = 0.48 [0.33 to 0.64] versus 0.47 [0.32 to 0.61]; immunology κ [95% CI] = 0.27 [0.14 to 0.41] versus 0.24 [0.12 to 0.35]). The κ scores calculated for IBV cases for PCR versus NAI (κ [95% CI] = 0.56 [0.43 to 0.69]) were higher compared to PCR versus HAI (κ [95% CI] = 0.24 [0.06 to 0.42]) in the serosurvey cohort but not in the immunology cohort. However, the κ scores were highest for PCR versus HAI or NAI (IAV = 0.33 to 0.48, IBV = 0.53 to 0.60). Thus, HAI and NAI assays have comparable sensitivities for IAV, but the NAI assay was more sensitive for IBV (sensitivity score [95% CI] = 1.00 [0.81 to 1.00]) compared to HAI (sensitivity score [95% CI] = 0.35 [0.17 to 0.56]). All assays had high specificity scores (range, 0.81 to 1), although, unsurprisingly, the combination of HAI and NAI assays offered the highest specificity (0.91 to 1).

The HAI and NAI assays also detected 4 and 20% seroconversions against H1N1 strain, respectively, in the serosurvey cohort and 1 and 13% seroconversions, respectively, in the immunology cohort (Table 5).

**TABLE 5:** HAI and NAI antibody responses to influenza A (H1N1) in the serosurvey and immunology cohorts

Virus	Age (yr)	Antibody titer^*a*^	Serosurvey cohort						Immunology cohort					
			*n*	HAI	95% CI	NAI	95% CI	By HAI/NAI (%)	*n*	HAI	95% CI	NAI	95% CI	By HAI/NAI (%)
Influenza A	0–4	GMT 1	9	29	–58–117	17	–54–88		6	10	10	5	5	
		GMT 2		37	–48–122	25	–109–160			14	–9–37	9	–42–59	
		GMFC		1	1–2	2	–0.02–3			2	–0.8–4	2	–8–12	
		No. (% SC)		1 (11)		1 (11)		22		1 (17)		1 (17)		30
	5–19	GMT 1	7	24	–3–52	108	16,200		4	8	6–11	57	34–79	
		GMT 2		24	–17–66	160	6–314			7	4–10	40	15–64	
		GMFC		1	1	2	1–2			1	0.2–2	1	1	
		No. (% SC)		0 (0)		1 (11)		11		0 (0)		0 (0)		0
	20+	GMT 1	9	22	2–41	93	–99–285		59	20	1–40	42	–60–144	
		GMT 2		23	2–45	93	–75–261			19	2–37	54	–288–397	
		GMFC		1	1	1	1			1	1	1	1–2	
		No. (% SC)		0 (0)		0 (0)		0		0 (0)		7 (10)		10
Influenza B	0–4	GMT 1	8	57	–33–146	57	–17–130		1	10	NA^*b*^	5	NA	
		GMT 2		62	–27–151	80	–70–230			10	NA	5	NA	
		GMFC		1	1	1	–0.3–3			1	NA	1	NA	
		No. (% SC)		0 (0)		1 (13)		13		0 (0)		0 (0)		0
	5–19	GMT 1	8	52	–39–142	247	–340–834		1	10	NA	80	NA	
		GMT 2		57	–34–148	269	–121–659			5	NA	40	NA	
		GMFC		1	1	1	1			0.5	NA	0.5	NA	
		No. (% SC)		0 (0)		0 (0)		0		0 (0)		0 (0)		0
	20+	GMT 1	10	49	–12–111	121	–124–366		23	11	1–21	45	–821–911	
		GMT 2		53	–22–128	279	–48–572			12	–2–27	65	–798–927	
		GMFC		1	1	2	48–52			1	1	1	1–2	
		No. (% SC)		0 (0)		2 (20)		20		0 (0)		2 (9)		9

a GMT, geometric mean titer; GMFC, geometric mean fold change. The percent seroconversion (%SC) was determined as the percentage of individuals that seroconverted, as defined by a 4-fold rise in antibody titer by the specified assay between the first and second sera.

b NA, not applicable.

### Specificity of assay in the control (summer) cohort.

To further examine the specificity of the assays, we tested paired sera collected for the diagnosis of nonrespiratory illnesses during the months when influenza activity was not epidemic. Our rationale for this was that any seroconversions detected were more likely due to assay complications rather than true infections. The percentages reported here represent seroconversion events against either IAVs (H1 and H3 subtypes) or IBVs (Yamagata and Victoria lineages) (Table 6). As a group, no significant rise in GMFC was detected for any virus. The frequency of a HAI-seroconversion event against IAVs, was 3% (95% CI = –1 to 6%) (3/94). All of these seroconversion events were directed against H3. The frequency of NAI seroconversion against IAVs was 4% (95% CI = 0 to 8%, 4/94). The frequency of NAI seroconversion against IBV was 2% (95% CI = 0 to 5%, 2/94), and no HAI seroconversion events were detected. Notably, three of the five individuals seroconverted to multiple antigens. Two individuals seroconverted to both N1 and N2, and one individual seroconverted to three antigens; H3 and NA to both B lineages (Table 7). No individual seroconverted to both HA and NA simultaneously.

**TABLE 6:** HAI and NAI antibody responses to influenza A (A/H3 and A/H1pdm09) and B (B/Yam and B/Vic) viruses in the non-influenza season (summer) cohort^*a*^

Type	Total no. of reactions^*b*^	Antibody titer^*c*^	HAI		NAI	
			*n*	95% CI	*n*	95% CI
Influenza A (H1 and H3)	94	First sera GMT	17	14–22	39	28–54
		Second sera GMT	18	15–23	43	31–60
		GMFC	1	0–2	1	0–2
		Seroconversion (%)	3	–1–6	4	0–8
		No. of individuals that seroconverted	3		2	
Influenza B (Yam and Vic)	94	First sera GMT	11	9–13	905	707–1,159
		Second sera GMT	11	9–14	967	767–1,220
		GMFC	1	0–2	1	0–2
		Seroconversion (%)	0		2	0–5
		No. of individuals that seroconverted	0		1	

a Summer in the Southern Hemisphere: December 2015 to March 2016.

b A total of 47 paired samples were tested against two influenza A or two influenza B virus strains, making a total of 94 reactions for each virus type.

c The percent seroconversion refers to the percentage of reactions that showed a 4-fold increase in antibody titer as determined by the specified assay between the first and second sera.

**TABLE 7:** HAI and NAI-antibody titers of the first and second serum sample in the five individuals who met the seroconversion criteria from the summer cohort

Antigen^*a*^	Sera	Individual				
		1	2	3	4	5
IAV_H1	First	40	5	5	10	5
	Second	40	5	20	10	5
IAV_H3	First	**10**	10	**5**	5	**10**
	Second	**40**	10	**40**	5	**40**
IAV_N1	First	80	**20**	320	**10**	320
	Second	160	**80**	320	**80**	320
IAV_N2	First	40	**10**	40	**10**	20
	Second	40	**80**	40	**40**	40
IBV (Victoria)_HA	First	5	80	5	5	5
	Second	5	80	5	5	5
IBV (Victoria)_NA	First	640	2,560	**40**	320	640
	Second	640	2,560	**160**	320	640
IBV (Yamagata)_HA	First	5	80	5	5	40
	Second	5	80	5	5	40
IBV (Yamagata)_NA	First	640	2,560	**40**	320	320
	Second	640	2,560	**160**	320	2,560

a IAV, influenza A virus; IBV, influenza B virus. Seroconversion events are indicated in boldface.

## DISCUSSION

Our study evaluated the induction of HA and NA antibodies in individuals with community-acquired influenza through two cohorts with a distinct sampling design. We found that the dynamics of HA and NA antibody responses were age and virus dependent after influenza virus infection. HAI seroconversion events in IAV cases were highest in young children (≤5 yo) but then decreased with increasing age. These young children, who are likely experiencing their first influenza virus infection, were also more likely to seroconvert to both HA and NA or show an HA-dominant antibody response. The latter finding is consistent with the expectation of HA being immunodominant compared to NA in HA-naive individuals (19, 20). The age-dependent increase in baseline NAI, but not HAI, titers in our cohorts suggests either an age-associated shift in antibody immunodominance or (perhaps more likely) a closer match between the NA antigen used in our assay to the prior circulating strain (i.e., a less-drifted NA antigen) (12). This could explain the NA-only seroconversions seen in IAV- infected adults. The presence of NA-specific memory B cells could favor the NA-specific antibody responses over HA in these adults, a model previously proposed to explain the “damping” of NA antibody responses in HA-primed individuals (21–23). Several studies have reported that the elderly subjects may have a more NA-biased antibody response compared to the younger subjects and that evidence of original antigenic sin can be observed for NA antibody responses as well (12, 24, 25). Based on these observations, it appears that NA antibodies to strains encountered early in life may persist and, like HA antibodies, can be “back-boosted” (26). It is therefore likely that the relative conservation of this antigen can further contribute to boost the preexisting NA antibody response in the adults.

Compared to the NAI assay, the HAI assay was surprisingly insensitive in detecting seroconversions against IBVs. This was also observed in our larger serosurvey cohort comprising of the PCR-negative cases (*n* = 701), where the proportion of IBV NAI seroconverters was higher than the HAI seroconverters, particularly in young children (27). This is in contrast to the findings by Rajendran et al., who reported lower postvaccination NAI titers to IBV in children compared to adults (12), suggesting that, like IAV, there are differences in the NA antibody response after IBV infection versus vaccination (28). Whether the reduced IBV HAI sensitivity seen in our study was due to technical reasons (i.e., a mismatched antigen or the influence of egg-adapted antigens) or antigenic competition, we found that the NAI assay compensated for these limitations, since 100% of the IBV cases were captured.

That the ELLA was particularly sensitive for IBV suggests that NA antibodies against IBVs could be more broadly cross-reactive than HA antibodies, as had been reported recently for IAV (28). However, we cannot exclude the possibility of nonspecific inhibition of NA activity by HA antibodies, particularly those that bind outside of the globular head which were not detected by HAI assays (since most of our participants had very low preexisting HAI titers to IBVs) (29, 30). How many of these antibodies are present and thus the extent of interference in polyclonal serum are unknown.

The two sampling designs used in the serosurvey and immunology cohort are typical of most seroepidemiology and clinical studies. The serosurvey cohort utilized a pre- and post-influenza season sampling design. This allowed for baseline sera to be collected prior to the actual infection, although with such a design, a large initial cohort is required in order to capture sufficient infected cases during the influenza season. In contrast, the immunology cohort sampled individuals that actively sought health care. Correspondingly, there was a large variability in the time of the first sera sampled after symptom onset in this cohort, attributed in part to the logistical delay in sampling the influenza-like illness (ILI) cases that present to the general practitioners across Auckland. This likely contributed to the high baseline titers, thus decreasing the likelihood of meeting the seroconversion criteria (31). Indeed, when we examined in detail the sampling times broken down by age (Table 8), we found that the older-children group (5 to 19 yo), which had the highest baseline HAI titer in the immunology cohort, was also the group that had the largest time interval between symptom onset and first serum collection. This is likely associated with health-seeking behavior since older children are less likely to present with severe influenza. We attempted to correct for the variable sampling time in our analysis by excluding the outliers (i.e., those sampled <14 days or >20 days), but this resulted in the loss of statistical power without affecting the population average (data not shown). Hence, we kept the original analysis for this study. Nevertheless, despite the difference detected in the frequency of seroconversions, data from both cohorts, at least for IAV cases, suggested that adults were more likely than children to show discordant HA and NA antibody induction, confirming previous observations (32, 33).

**TABLE 8:** Details on the time interval between the first serum collection from symptom onset or swab specimen and the times between serum collection, as stratified by age and ethnicity, in the serosurvey and immunology cohorts^*a*^

Characteristics	Serosurvey cohort (*n* = 50)					Immunology cohort (*n* = 94)				
	*n*	%	Median days (range)			*n*	%	Median days (range)		
			A	B	C			A	B	C
Age (yrs)						
0-4	16	32	128.5 (53–180)	134.5 (63–188)	220 (132–259)	7	7	7 (1–14)	2 (0–6)	17 (14–24)
5-19	15	30	142.5 (22–193)	147 (28–176)	215 (133–249)	5	5	17 (10–28)	12 (7–23)	14 (12–19)
20+	19	38	137.5 (29–170)	144.5 (35–178)	216 (144–274)	82	87	12 (1–26)	6 (0–19)	16 (5–27)
Sex						
Male	16	32	113 (29–193)	118 (35–185)	216.5 (139–274)	44	47	12 (1–28)	5 (0–23)	17 (5–27)
Female	34	68	141 (22–180)	148 (28–188)	218.5 (132–259)	50	53	13.5 (1–26)	6 (0–15)	14 (12–24)
Ethnicity						
Maori	7	14	137.5 (105–165)	148.5 (113–169)	232 (207–259)	15	16	12 (1–18)	5 (0–13)	16 (12–24)
Pacific	5	10	79.5 (22–113)	87.5 (28–118)	210 (132–274)	23	25	11 (5–14)	6 (1–10)	15 (14–27)
Asian	9	18	141.5 (109–163)	148 (115–167)	214 (144–250)	8	9	16 (12–17)	5.5 (2–12)	16 (14–21)
European-descent/Other	29	58	138.5 (29–193)	144.5 (35–188)	219 (139–249)	47	51	12.5 (1–28)	6 (0–23)	16 (5–24)

a Time intervals in the table are abbreviated as follows: A, time from first serum collection to symptom onset; B, time from first serum collection to swab specimen; and C, time between serum collections.

Despite H3N2 being the dominant circulating strain during our study, some seroconversions to H1N1 were also detected in our two cohorts. Seroconversions to NA were more frequent compared to HA, potentially due to the higher cross-reactivity of NAI antibodies or to coinfection events during the influenza season. For this reason, we used the summer cohort (where sera were collected for nonrespiratory disease testing when community influenza activity is low) as a proxy for an “influenza-negative” population to evaluate the specificity of these assays. Since three of the five paired sera showed seroconversion to multiple antigens (Table 7), it is likely that these are false-positive events. With the caveat that we could not discount the possibility of any “out-of-season” influenza cases, the false-positive frequencies were between 0 and 4% of the total reactions tested and appeared to be comparable between HAI and NAI assays.

Principally, we found that including the NAI assay improved the serological detection of influenza cases in both our cohorts, although we acknowledge that the detection sensitivities of the HAI and NAI assays reported here may vary during different influenza seasons with different antigens. With further validation across different influenza seasons and antigens, including the NAI assay as a hierarchical or targeted testing approach can increase the power and accuracy of a seroepidemiological study and will circumvent some of the limitations and variable robustness associated with HAI assay.

Overall, our findings highlight the age and subtype-dependent nature of HA and NA antibody responses after PCR-confirmed influenza virus infections. The differences in the antibody response to HA and NA that we observed between adults and children suggests a possible influence of immunological memory on the recall response. This is an important consideration for any vaccination strategy aimed at eliciting NA immunity since it suggests that the antibody response may differ with different age groups. The mechanism underlying such response dynamics should be further investigated.

## MATERIALS AND METHODS

### Ethics approval.

This study received ethics approval from the New Zealand Northern (A) Health and Disability Ethics Committee under references NTX/11/11/102/AM02, AM05, AM06, AM13, and AM14.

### Study design.

The sampling designs for this study are summarized in Fig. 1. For the serosurvey cohort, randomly selected community participants enrolled at SHIVERS participating general practices provided pre-influenza season sera in March to June and post-season sera in October to November of 2015. During the influenza season (May to September), nasopharyngeal swabs from 209 unvaccinated participants with influenza-like illness (ILI; defined as cough and fever with onset ≤10 days) were tested by PCR for evidence of influenza virus infection. Fifty-one infections (24% positivity) were identified, and the sera from these individuals were analyzed for this study. The immunology cohort was recruited in the 2013 and 2015 seasons and consisted of 94 patients presenting to general practice clinics (*n* = 29) with acute respiratory illness or to hospitals with severe acute respiratory illness (*n* = 65) based on the World Health Organization disease classification (34). For this cohort, the first blood draw occurred following a positive influenza PCR test, and the second occurred at least 14 days later. Study data were collected and managed using Research Electronic Data Capture (REDCap) tools (35) hosted at the Institute for Environmental Science and Research (ESR). To examine the specificity of the assays, a panel comprising of 47 paired sera, collected approximately14 days apart during the summer of 2016 for arbovirus/legionella testing at the ESR, was also evaluated.

### PCR diagnosis.

Respiratory samples were tested and subtyped using the U.S. Centers for Disease Control and Prevention real-time RT-PCR protocol (16) or the AusDiagnostics PCR protocol (36), as previously described (37–39).

### Serologic testing.

Serologic testing was performed at the National Influenza Center at ESR, New Zealand. Receptor-destroying enzyme (RDE)-treated sera were tested against the strains included in the Southern hemisphere vaccine for the relevant enrollment year (A/H3, A/H1, and the two B lineages; Table 9) according to standard practice (40). The ELLA was used to test for NAI-specific antibodies, as previously described (9, 11, 41). Recombinant influenza A viruses (IAVs) composed of NA from the viruses to be tested and a mismatched HA from A/Teal/Hong Kong/W312/1997 (H6N1) were generated using the reverse-genetics method and used as antigens. The NAs used in recombinant viruses were N1 from A/California/04/2009 (H1N1) and N2 from A/Victoria/361/2011 (H3N2) and A/Switzerland/9715293/2013 (H3N2). Wild-type viruses B/Brisbane/60/2008 (Victoria lineage [Vic]) or B/Phuket/3073/2013 (Yamagata lineage [Yam]) (9) were used in the influenza B virus (IBV) ELLA. Serum samples were tested at a starting dilution of 1:10. For the summer control cohort, sera from those individuals were tested against all four influenza viruses, and the percentages of seroconversion events against IAV (A/H3 and A/H1) and IBV (B/Yam and B/Vic) were reported.

**TABLE 9:** Virus strains tested for each cohort

Yr	Subtype	Virus strain	Cohort(s)
2013	H1N1	A/California/7/09	Immunology
	H3N2	A/Victoria/361/2011	Immunology
	B (Victoria)	B/Brisbane/60/2008	Immunology
	B (Yamagata)	B/Massachusetts/02/2012	Immunology
2015	H1N1	A/California/7/09	Serosurvey, immunology, and summer
	H3N2	A/Switzerland/9715293/2013	Serosurvey, immunology, and summer
	B (Victoria)	B/Brisbane/60/2008	Serosurvey, immunology, and summer
	B (Yamagata)	B/Phuket/3073/2013	Serosurvey, immunology, and summer

### Statistical analysis.

Seroconversion was defined as a 4-fold increase between the first and second HAI or NAI titer against the subtype of the infecting virus. In addition, if the first titer was below the detection threshold (<10), the second titer had to be ≥40. IAV cases that were not subtyped were assumed to be A/H3N2, since it was the predominant subtype circulating in both seasons of the study. Non-lineage-typed IBVs were assumed to be the B antigens with the highest increase in titer. Geometric mean titers (GMTs) were reported as the back-transformed average of the log_2_ antibody titers, and the geometric mean fold change (GMFC) was reported as the back-transformed average of the differences between the first and second log_2_ antibody titers (41). Age-specific effects on the antibody responses were analyzed by using analysis of variance (ANOVA) tests.

Kappa (κ) statistics were used to describe the level of agreement between PCR diagnosis and seroconversion status by HAI only, by NAI only, by both HAI and NAI, and by either HAI or NAI. The strength of agreement between the two assays was categorized based on the Landis and Koch kappa benchmark scale (23). Test sensitivity and specificity scores were also calculated for each assay or combination of assays. For the serosurvey cohort, the negative cases were the ILI individuals that were PCR negative during the season (*n* = 209). For the immunology cohort, the negative cases were derived from the summer cohort. For IAV, we considered PCR subtype positivity as the gold standard outcome, and we used the homologous subtype seroconversion event as the test to ascertain agreement. For IBV, we only considered PCR type and any flu B serotype seroconversion for agreement. All calculations were generated by using the R package epiR (42, 43).
